# Through the Eyes of Children: Perceptions of Environmental Change in Tropical Forests

**DOI:** 10.1371/journal.pone.0103005

**Published:** 2014-08-05

**Authors:** Anne-Sophie Pellier, Jessie A. Wells, Nicola K. Abram, David Gaveau, Erik Meijaard

**Affiliations:** 1 Center for International Forestry Research (CIFOR), Bogor, Indonesia; 2 ARC Centre of Excellence for Environmental Decisions, Centre for Biodiversity & Conservation Science, University of Queensland, Brisbane, Australia; 3 Durrell Institute for Conservation and Ecology, School of Anthropology and Conservation, Marlowe Building, University of Kent, Canterbury, Kent, United Kingdom; 4 Borneo Futures initiative, People and Nature Consulting International, Jakarta, Indonesia; Universidade de São Paulo, Brazil

## Abstract

This study seeks to understand children's perceptions of their present and future environments in the highly biodiverse and rapidly changing landscapes of Kalimantan, Indonesian Borneo. We analyzed drawings by children (target age 10–15 years) from 22 villages, which show how children perceive the present conditions of forests and wildlife surrounding their villages and how they expect conditions to change over the next 15 years. Analyses of picture elements and their relationships to current landscape variables indicate that children have a sophisticated understanding of their environment and how different environmental factors interact, either positively or negatively. Children appear to have landscape-dependent environmental perceptions, showing awareness of past environmental conditions and many aspects of recent trends, and translating these into predictions for future environmental conditions. The further removed their present landscape is from the originally forested one, the more environmental change they expect in the future, particularly declines in forest cover, rivers, animal diversity and increases in temperature and natural disasters. This suggests that loss of past perceptions and associated “shifting environmental baselines” do not feature strongly among children on Borneo, at least not for the perceptions we investigated here. Our findings that children have negative expectations of their future environmental conditions have important political implications. More than other generations, children have a stake in ensuring that future environmental conditions support their long-term well-being. Understanding what drives environmental views among children, and how they consider trade-offs between economic development and social and environmental change, should inform optimal policies on land use. Our study illuminates part of the complex interplay between perceptions of land cover and land use change. Capturing the views of children through artistic expressions provides a potentially powerful tool to influence public and political opinions, as well as a valuable approach for developing localized education and nature conservation programs.

## Introduction

Environmental change typically occurs over long, decadal, time frames. Deforestation, pollution of water ways, or declines in species numbers often take place gradually, and notable changes are only apparent once certain thresholds are passed, for example, when species become extinct, or pollution creates significant health problems [1]. Gradual environmental changes can lead to inter-generational differences in perceptions of environmental conditions, often referred to as the shifting baseline syndrome [2], [3]. Shifting baselines have been extensively demonstrated in perceptions of fisheries [3], [4], [5], [6], but this phenomenon also occurs in terrestrial environments, impacting conservation management and influencing environmental policy-making [7]. For example, if perceptions of the former abundance of threatened species, such as the orangutan (*Pongo pygmaeus*), are lost over time, there may be little public or governmental support to enable populations to recover to ecologically optimal densities. Similarly, people's perceptions of what constitute forests can change, if people forget what forests were once like, or have adapted their perception of forests to their present reality [8]. These altered perceptions can impact conservation efforts, if they result in lack of public support for conserving forest ecosystems or protecting areas of remnant, intact forest from loss or degradation.

Shifting baseline perceptions are of particular concern among children and the younger generations of a community, as it is they who will ultimately play the biggest role in determining future use and management of the natural environment [2], [9], [10]. Compared to older generations, however, children have a shorter term association with past environmental conditions and are thus more likely to use present-day contexts as their reference framework. For example, a child who has only experienced current degraded environments might be unaware of earlier, more natural conditions, or of the potential for recovery to pre-disturbance conditions. This shift is more likely to occur with a lack of communication between generations and/or of information on past (and current) environment conditions [4], [7]. Most children have longer lives ahead of them than adults and thus greater stakes in future environmental and social conditions, which will influence their long-term well-being. Children are also engaged in rapid learning and have fewer pre-conceptions than older generations. They may therefore be the ideal group for targeting conservation education and awareness programs that aim to increase consciousness of how natural ecosystems and biodiversity are changing, and motivate more sustainable interactions with their environment.

In this study we focus on Indonesian Borneo, South-East Asia, a hotspot of endemism and biodiversity [11]. These biologically rich environments are, however, under increasing threat, as Borneo has some of the highest rates of deforestation and forest degradation in the world [12], [13]. Between 1973 and 2010, at least 168,493 km^2^ (30.2%) of closed-canopy forests were cleared across the island, and a further 179,917 km2 (32%) were logged [14]. By 2010, Borneo's land cover consisted of 28% intact forests, 24% logged forests, 10% industrial plantations, and the remaining 38% included secondary forests, agroforests, grasslands, croplands and other land uses [14].

Governments in Borneo are principally democratically elected and political decision-making can be influenced by peoples' opinions. Therefore, capturing perceptions about land use and forest values, and understanding what drives spatial variation within these perceptions, offers important knowledge for spatial land use planning and natural resource policies. The Borneo Futures initiative, under which the present study was conducted, was established to perform these assessments, and has led to new insights about how people view their relationships with forests and other land uses, how this varies across the large and culturally diverse island, and what environmental and social factors underlie these perceptions [8], [15], [16], [17]. These findings are vital if land use conflicts are to be minimized, environmental health restored, biological diversity conserved, and sustainable economic development enhanced.

In this paper, we build an additional dimension to the Borneo Futures initiative by focusing on children's perceptions of forest landscapes and wildlife surrounding villages in Kalimantan, Indonesian Borneo. Specifically, we assess children's perceptions of the current and future state of their environment, and whether these perceptions differ among children who are growing up in different landscape contexts: forested landscapes, recently deforested landscapes, or old agro-forestry landscapes. To assess this, we employ a novel approach by using children's drawings to gain insights into their perceptions of environmental change. Although other studies have used similar techniques for studying children's thoughts [18], [19], [20], to our knowledge this is the first study to use this approach to assess perceptions of environmental change across dynamic forest environments.

## Materials and Methods

### Ethics statement

#### Institutional and authority level

The surveys of children's drawings were conducted in parallel with a broader set of interview-based surveys of adult villagers regarding ecosystem services and wildlife [8], [15], [16]. Written approval for our survey was given by the Indonesian State Ministry of Research and Technology (RISTEK) under their research agreement with the Center for International Forestry Research (CIFOR). Prior to commencing field work in each district, the researcher registered the project's methods and purpose with district government officials. At the village level, approval by local authorities was given verbally by the village head or other senior village leader at the time of the research. As CIFOR has neither an ethics committee nor institutional review board, the proposed methodology was discussed with CIFOR's senior social scientists who gave us verbal approval to proceed, pending each potential research subject's free, prior and informed consent to participate in the research. This consent required us to give villagers, teachers and children sufficient information about the study's design and objectives so that they could make a free and informed decision about whether they agreed with the research occurring in the village, and specifically whether they wished to participate.

#### Village level

In each village, the consent process involved meetings with the village head and village leader, followed by a community meeting with school teachers and other adult members of the village, including the parents of potential participants. During these meetings a standard statement in Indonesian was read out (and provided in hard copy) which specified the identities of the researcher and her field assistant, the purpose and value of the research, and that each child's participation was to be voluntary and anonymous. We outlined that children would be asked to draw the environment around their village, with features such as the forest and animals. (More specific details of the drawing activity and its request to consider the environmental conditions in 15 years' time were given on the day of the activity, not during the village meeting, because we did not want the children's views to be influenced by ourselves or by others in the village before the time of the activity). We specified that we were seeking children between 10 and 15 years old to participate, and that younger children (7–9 years) would be invited to participate if fewer children in the target range were present. We requested verbal consent to use the drawings in research, workshops, magazines and any research papers, and on a web platform. We assured villagers that the data would be treated confidentially, and neither the names of villages nor the names of participants would be publicized with these drawings. An additional meeting with the school head and teachers on the morning of the research activity was held providing information on the aims of the study and requesting their verbal confirmation of their consent. In the classroom, the children were clearly informed of the identity of the researcher and the details of the drawing activity (for example, that they would each join a group of children and draw a picture together, which would show either how they saw their present environment, or their expectations for their environment 15 years in the future). We emphasized the voluntary nature of participation and that non-participation would not affect them negatively, and assured anonymity of their names and their village. Finally, children were asked to verbally confirm that they understood this explanation, then asked to freely choose to participate or not. Those who chose not to participate were free to leave the classroom. After completion of the drawings, each participant was asked to write his or her first name on the reverse side of the drawing sheet (or the first letter of his/her first name if he/she was not able to write), to indicate their voluntary participation in the activity, noting that their name would remain anonymous and would not be visible in any display or in any data sheet analysis of the research.

#### Reasons for verbal consent

We did not obtain written consent for two reasons. Firstly, there is a common reluctance by people in Indonesia to sign documents due to fears of their use by authorities to their detriment. Secondly, the resulting lag time between introducing the research topic to villagers and the actual implementation of the drawing studies would have created significant opportunities for children's perceptions to be influenced by their parents, teachers or each other. As the primary goal of the study was to capture the children's own perceptions, it was essential to minimize such influences. Formal written consent would have required parents to sign on behalf of their children, resulting in time lags of several days because of the logistical difficulty of visiting people who are often working in fields away from the village.

### Data Collection

**Figure 1:**
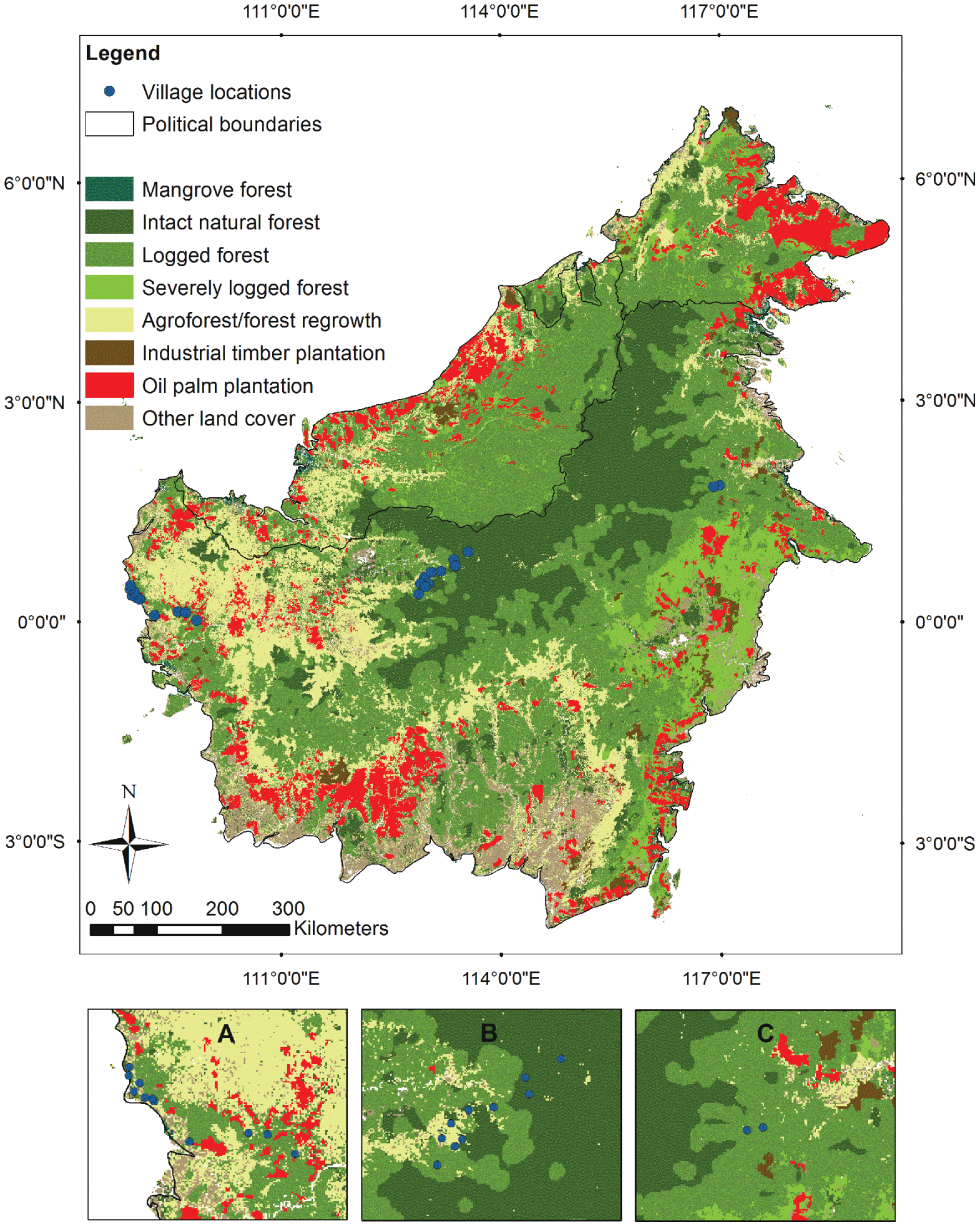
Surveyed villages in Kalimantan, Indonesian Borneo with land cover (2010) classes. The map shows the land cover and land use across Borneo island in the year 2010 (see Figure legend, and methods for sources), and the locations where the children's drawing surveys were conducted in twenty-two villages (blue dots) in three different parts of Kalimantan, Indonesian Borneo. Insets: (A) Ten villages at the lower Kapuas in West Kalimantan Province; (B) Ten villages at the upper Kapuas in West Kalimantan Province; (C) Two villages at the upper Kapuas in East Kalimantan Province.

We chose three broadly different environmental settings for the surveys: 1) an area dominated by old agroforestry and plantations (mostly for palm oil and rubber production) in the lower Kapuas River region in West Kalimantan Province; 2) an area dominated by old growth forests and slash-and-burn agriculture in the upper Kapuas River region of West Kalimantan; and 3) a remote area dominated by selectively logged natural forest in the upper Kelay River area in East Kalimantan (Figure 1). Without having prior knowledge about the villages, we randomly selected 22 villages, consisting of 20 in West Kalimantan and two in East Kalimantan.

In each village, classes were composed of children from a range of ages, where often the age of a child could not easily be linked with their level of school education, especially in the more remote villages. If there was no school in the village or if the visit occurred during school holidays, children were asked by the village head whether they wanted to participate. In the classroom, children were asked to join one of two groups to work together on one drawing per group. These groups consisted of both boys and girls mostly aged between 10 and 15 years old (see Table 1). We targeted this age range because our pilot study had revealed that the aim of the activity was clearly understood by children in this age class, whereas younger children sometimes found it difficult to understand what they were asked to draw. In some villages, where the number of children in the target age range was less than six per group, younger children aged 7, 8 and 9 years old joined the activity. The children of different ages were allocated evenly between groups, to ensure that the age ranges for the groups drawing the present and future were as similar as possible. The ages of the children were then confirmed by their teacher or head of school. Groups were allocated in different parts of the classroom to minimize interactions between them.

**Table 1:** Ages and numbers of children who participated in drawing surveys in 22 villages in Kalimantan.

Drawing time period	Number of villages	Average number of children	Average age of children	Male	Female	Total of children*
**Present**	22	6	11.4	73	72	145
**Future**	22	6	12.0	65	76	141
**Totals or Mean**	44 drawings, 2 per village	6	11.7	138	148	286

*Note: In four of the villages, 39 children participated in both the present and the forest future drawing sessions, however, if a child had already drawn, then he or she only participated in giving ideas for the second drawing. Consequently, a total of 247 children participated in the drawing study with 131 girls and 116 boys from 22 different villages.

Within each village, the average age of children in the ‘present’ and ‘future’ drawing groups were as similar as possible.

### Drawing surveys

In each village, we asked one group to draw the current condition of the forest and the animals they would expect to find in the forest. The second group was asked to draw their vision of the forest and animal life in the forest in 15 years from now. To clarify the 15 year time frame, we referred to a period when participating children would be adults and may have their own children. We reminded the children that it was not important how skillful they were at drawing and that the important thing was to draw what their group really thought about present or future conditions of forests. Children were encouraged to discuss ideas within their group before committing them to paper. Sometimes children of the same group had different ideas, and emphasis was given for them to either draw a compromise idea or to include all the different ideas in their drawing. No time restrictions were imposed. The average time spent was two hours, and in all cases the children reported that they completed their drawing. We requested that during the activity the teacher would not interact with the children, discuss their drawings or provide additional explanation. Also, no books or images were allowed for the activity, to ensure that we captured the children's unbiased perceptions. During the drawing exercises, author ASP also observed the discussion and drawing processes and took notes about particular decisions the children made.

### Drawing interpretations

Once the drawings were complete, each group was asked to describe their drawings and these explanations were recorded on video and later used to verify particular features of the drawings and gain further insights into their perceptions. These interviews lasted until the children had nothing more to say about their drawing. If some elements were still unclear, we asked children for clarification through some specific questions regarding the amount of forest left in their landscapes, and its current and expected future conditions. Additionally, we asked whether the forests they had drawn contained many, few or no animals, and which animals occurred or had disappeared. Communication during the surveys was primarily in Indonesian. In remote areas, children understood Indonesian but were more comfortable in their local language. Many items in the drawings are captioned in these different local languages and we asked for input from both children and the school teachers to translate them into Indonesian.

### Survey data management

All of the elements depicted by children in their drawings and described in their writings, and all oral comments were entered in a database. We assigned features to eight categories (from which six were used for analysis, see Table 2), with some categories following Bowker [18]: 1) Broader landscape features (e.g., mountains, sun, sky, clouds, rainbow, rain, temperature condition, rivers, waterfalls, and lakes); 2) Botanical diversity (e.g., frequency and number of each different type of trees and plants in either natural forest or agro-forest: fruit trees, hardwood trees, medicinal plants, resinous trees, other tree types, shrubs, flowers, vines, fungi and grassland, and total of this vegetation diversity (sum of different types)); 3) Faunal diversity (e.g., the frequency and number of different animal species and general condition of animal wildlife); 4) Forest condition features (e.g. including village-forest distance, areas of continuous-canopy forest (not being cleared) and disturbed forest areas, and the presence of felled trees); 5) Agricultural features (e.g., oil palm trees or plantations, rice fields, and rubber plantations); 6) Features relating to other environmental issues (natural and anthropogenic) (e.g., natural disasters, such as floods, landslides, erosion, and forest fires; condition and pollution of rivers; and elements related to development, including roads, vehicles, hotel buildings, large housing, factories, electricity poles/towers, etc.); 7) People in the forest or village (e.g., the number of rural people in the forest or in the village, their location, their activity, boat types, and equipment); and 8) Village features (e.g., village occurrence in the drawing, number of houses, and any other building types such as churches, schools, and traditional monuments).

**Table 2:** Environmental art variables depicted in drawings by children from 22 villages in Kalimantan.

Category	Environmental variables from drawings	Code used in figures
**Landscape**	**Condition of mountain forests**	**For.Mtain**
	**Temperature condition**	**Temp**
	**River condition**	**RiverC**
**Botanical diversity**	**Vegetation diversity** (Total number of vegetation types, excluding grassland)	**VegE**
**Faunal diversity**	**Faunal condition - Abundance and diversity of wildlife**	**FaunaC**
	**Threats to animal wildlife** (9 individual threats coded from 1 to 9: a single code value was assigned to each drawing; if several threats occurred in a given drawing, a single code value was assigned as the addition of the specific threats)	**ThreatA**
**Forest condition**	**Village-Forest Distance** (areas of non-felled trees and agro-forest trees if represented in forests)	**ViForD**
	**Disturbed Forest areas** (Shown as location of logged, burned or degraded forests; e.g. ‘only in mountain part’; ‘surrounded village’; ‘both mountainous areas and surrounding the village’)	**Defo**
	**Undisturbed Forest areas** (i.e. Location of forests not being cleared or degraded at this time; can be primary and/or secondary forests)	**NoDefo**
**Agriculture fields**	**Oil palm area cover**	**Poil**
**Other environmental issues (natural and anthropogenic)**	**Presence of industries**	**Ind**
	**Presence of main roads**	**Mroad**
	**People clearing the forest** (no clearance; few; a lot)	**MenClear**
	**Flood occurrence**	**FL**
	**Non-flood disasters occurrence**	**NFLdis**

We coded each individual element either as binary values (absence/presence of each element, or good/bad for the element's condition), as ordinal values (e.g., few, several, many; or, beside, close, far, far away), or as numerical counts of elements or element types such as particular species. In addition, some variables were assigned a set of further ‘conditions’ only as nominal values (e.g., ‘undisturbed forest areas’; ‘threats to animals’; etc.). Each of the measures or individual elements described above constitutes an ‘art variable’ for statistical analysis.

### Spatial variables

We assessed relationships between children's perceptions of forests and their village landscape contexts represented by 13 spatial variables consisting of land use and land cover (LULC), climate, topographical and socio-economic variables (see Table S1 for descriptions and abbreviations of spatial variables; sources and processing steps are outlined below, and are described in full in reference [8]).

We extracted values of these spatial variables for each sampled village by mapping village locations within a Geographic Information System (GIS), using the Global Positioning System (GPS) way points taken in the centre of each village.

Analysis of land use and land cover considered five LULC classes: intact natural forest; agro-forests/forest re-growth; oil palm plantations; other land cover types; and a general ‘forest cover’ layer developed by combining the intact forest and logged forest layers. The villages surveyed were surrounded mainly by ‘logged forest’, whereas intact forest was less extensive. The overall ‘forest cover’ was strongly positively correlated with the cover of ‘logged forest’, and weakly negatively correlated with ‘intact forest’ cover. The LULC spatial variables were derived from the integration of three datasets: firstly, a SarVision PALSAR 2010 (50 m resolution) classification, where classes were either used separately, or aggregated to form more general classes; secondly, logging road data networks (digitized from Landsat imagery from the years 1990, 2000 and 2002, [21]) were used to distinguish intact and logged forest classes, and to calculate a road density index (the length of logging roads and major roads within a 5 km radius of the village); and thirdly, digitized data sets of oil palm plantations developed through onscreen digitising of >150 Landsat images from the 1990-, 2000-, and 2010-eras, downloaded from the Global Land Survey database (http://earthexplorer.usgs.gov/) (for details see [21], [22]). For each LULC class, we calculated the Euclidean distance from the village to the nearest example of that class (in meters), such as the distance to the nearest oil palm plantations or to logged forest (see Table S1). For each of the five LULC classes, and for an additional class indicating protected area status [21], we also calculated the summed values of neighbouring cells within a 10 km radius (using focal statistics in ArcGIS 10.0). This gave a set of values for each village, detailing the summed cover of each LULC class and of protected areas in the landscape around the village. Secondly, the climatic variable ‘annual precipitation’ was included, along with elevation grid data from the WorldClim, ver. 1.4 dataset (www.worldclim.org) at 30 arc-seconds resolution. A river density index was generated by applying a kernel density tool in ArcGIS 10 to the Hydrosheds rivers dataset sourced from http://hydrosheds.cr.usgs.gov/index.php [23]. Thirdly, a human population density layer (i.e., estimated number of people per 1 km^2^) was derived from the LandScan 2007 dataset [24] and used to calculate ‘settlement density’ using a kernel density function for cells with 10 or more people per km^2^. All spatial data were developed at 30 arc-seconds (approximately 1 km^2^) resolution and projected. We then extracted all spatial data for each village location to use within the analyses outlined below.

### Statistical analysis

Art variables were first summarized using descriptive statistics. To compare the art variables between the present and future drawings we used contingency table tests (contingency coefficient (Cc)) for statistical differences in frequencies for each binary or nominal variable, and we used non-parametric Mann-Whitney (U) tests for differences in numerical (ratio-scale) and ordinal variables. To understand the relationships between variables, correlation coefficients were estimated for each pair of art variables and spatial variables using Pearson's correlation coefficient (*r_P_*) for ratio scale variables, and Spearman's rank correlation (*r_s_*) for pairs involving binary or ordinal variables. To summarize the current physical landscape of the villages, we conducted a principal component analysis on the 13 spatial variables in SPSS 17.0 (SPSS 2010) using the factor analysis function. We next conducted a categorical principal component analysis (CatPCA) of the art variables as a combination of numerical (ratio scale), ordinal, binary and nominal variables, and compared the multivariate axis scores between drawings of the present and the future. We also explored the relationships between the current land cover of the villages and the art variables describing the children's perceptions of current and future environmental conditions. In the CatPCA analyses, numeric variables that contain decimal values were converted to rank variables, to enable handling of values <1 (these variables consisted of the physical landscape layers and one art variable ‘Vegetation diversity’). All variables were given equal weight in the multivariate analyses.

## Results

### Drawings summary

**Figure 2:**
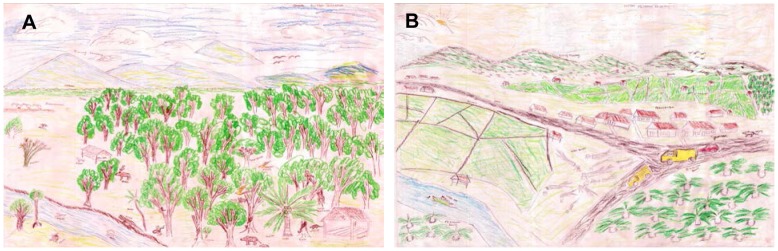
Drawings of the present and future by children in lower Kapuas, West Kalimantan. Both the drawings represent respectively the present (A) and future conditions (B) of the forest and animal wildlife. The children provided explanations in the drawing as the following: A) Present conditions: “The forest is an oxygen giver which is used to make a living from forest trees: rattan, rubber trees, durian, bamboo, etc. Forest is home to wild animals (e.g. snakes, wild cats, monkeys, pigs, birds, deer, squirrels, etc.) and among those there are several which live in the river (e.g. crocodile, fish and turtles). Nevertheless, wild animals are being hunted by humans and are thus disappearing (e.g. snakes, monkeys, deer, pigs, pangolins, birds, etc.). Animals that live around the village are chickens, pigs, dogs, and cats. The forest is still good, especially in the mountains.” B) Future conditions: “Forest is currently home to many different animals and to human livelihoods. In the future, the air that was fresh in the present time is getting warmer, including the mountainous regions. Nothing is green anymore and generally forest is replaced by oil palm even in the mountain where it only remains as few small trees. The number of trees decreases due to palm oil companies, factories, and other human activities such as illegal logging to build houses, mining for materials, and others for building highways. These changes will cause natural disasters such as erosion, landslides, and floods. The number of animals decreases, and little by little they will disappear, except dogs and chickens. The rivers (with water boats) are being polluted by scattered waste, and fish will be less abundant and will then go extinct.”

A total of 247 children participated in the drawing study (131 girls and 116 boys) from 22 different villages, resulting in 44 drawings (see Figure 2 for an example). The four animal taxa with highest representation in the drawings were: birds (22% of wild animals), mammals (21%), reptiles (21%), and fish (19%). Children often drew people in the forest engaged in some activity, such as fishermen in boats or farmers working in their field and gave several examples of different trees (fruit, resinous and timber trees), animal species, and forest products (e.g. rattan and honey). Sometimes children, especially those living in more densely forested areas, represented their cultural and spiritual beliefs relating to environmental features, for example by drawing a ‘*rumah hantu’* (house of ghosts) in the forest, or a dragon in the river. Overall we distinguished 180 different elements in the eight different categories. Fifteen art variables were selected for further analysis (see Table 2), consisting of individual elements or their summaries (e.g., total ‘vegetation diversity’ as the sum of plant types) (see below). These art variables were selected as the elements that appeared frequently in drawings from multiple groups, and had been specifically mentioned by children during their explanation of their drawings.

### Correlations between art variables within each time period

In the ‘present day’ drawings, children depicted a wide range of environmental conditions for most variables, such as the condition of forest near the village, faunal diversity, and vegetation diversity. Of the 15 variables, three were represented as being in ‘good’ conditions in all villages (‘temperature’, ‘forest mountain’, and ‘river’ conditions'), and hence showed no statistical variation (in contrast, these variables did show variation across drawings of the future).

Several of the art variables from ‘present’ drawings are strongly correlated (Table S2). For instance, ‘main road’ and ‘flood occurrence’, which are positively correlated to each other (r = 1.0, p<0.05 in all cases mentioned), are both similarly and positively correlated with the ‘presence of industries’ (r = 0.69) and ‘threats to animals’ (r = 0.65). The latest variable is also negatively correlated with ‘faunal condition’ (r = −0.79), while ‘vegetation diversity’ shows a negative correlation with ‘disturbed forest areas’ (r = −0.64).

The drawings and discussion of the ‘future’ showed variation across villages in the condition of almost all environmental features, with generally higher variance than across the ‘present’ drawings. All art variables showed variation, except for ‘people clearing the forest’, which was categorized as ‘many’ in the future for all villages. Correlations among ‘future’ art variables are shown in Table S3. Interestingly, ‘flood occurrence’ is significantly correlated with all other variables, except ‘oil palm cover’ and ‘threats to animals’. Though ‘flood occurrence’ is only weakly correlated with ‘main road’ presence (r = 0.42), they both show similar negative correlations with other art variables (conditions of ‘mountain forests’ (respectively, r = −0.65 and −0.68), of ‘rivers’ (resp., r = −0.47 and −0.46), and of ‘animals’ (resp., r = −0.48 and −0.50), ‘undisturbed forest areas’ (resp., r = −0.46 and −0.64) and the ‘vegetation diversity’ (resp., r = −0.45 and −0.49)); and positive correlations with ‘village-forest distance’ (resp., r = 0.46 and 0.56) and ‘disturbed forest areas’ (resp., r = 0.48 and 0.56). ‘Main road’ presence is also positively correlated with ‘oil palm cover’ (r = 0.43).

### Differences between present and future environments

Comparison between drawings of present and future conditions showed the following differences: local temperatures are expected to be higher in the future (p<0.001, U = 99.0); future conditions of mountain forests and rivers are expected to deteriorate (respectively: p<0.001, U = 19.0; and p<0.001, U = 50.0, for example 100% showing ‘good’ river condition in the present, compared to 58% ‘bad’ in the future), and the number of people clearing the forest is expected to increase (p<0.001, U = 66). A closer analysis of forest conditions indicates that distance between villages and the nearest forest will be much greater in the future (p<0.001, U = 54.5), with 22.7% of ‘future’ drawings showing the highest distance class, and 68.2% placing villages in areas that were previously, but no longer, forested. In the present, the ‘undisturbed’ forest areas occur around villages and on mountains (68.2%), though some villages showed this undisturbed forest only on the mountains (27.3%). In the future, undisturbed forests are expected to be more limited, either restricted to smaller areas (40.9%), or disappearing completely (40.9%) (p<0.001, Cc = 0.635). In contrast, the disturbed forest areas are much more widespread in future drawings. For example, the majority of ‘present’ drawings had few felled trees or deforested or degraded areas (63.6%), whereas all of the ‘future’ drawings contained deforested or degraded areas (p<0.001, Cc = 0.651). In the future drawings, disturbed forest was indicated by cleared and degraded areas drawn around the village and even on mountains (summed value: 72.7%), or by the complete absence of forest areas from the drawing (27.3%). Furthermore, felled trees appeared in 59.1% of present drawings compared to 95.5% of the future drawings (p = 0.004; Cc = 0.398). The presence or absence of oil palm plantations did not differ significantly across the two time periods, however, the size of oil palm areas drawn in the ‘future’ pictures was much greater than in any drawings of the ‘present’ (p = 0.01, U = 0.0).

**Figure 3:**
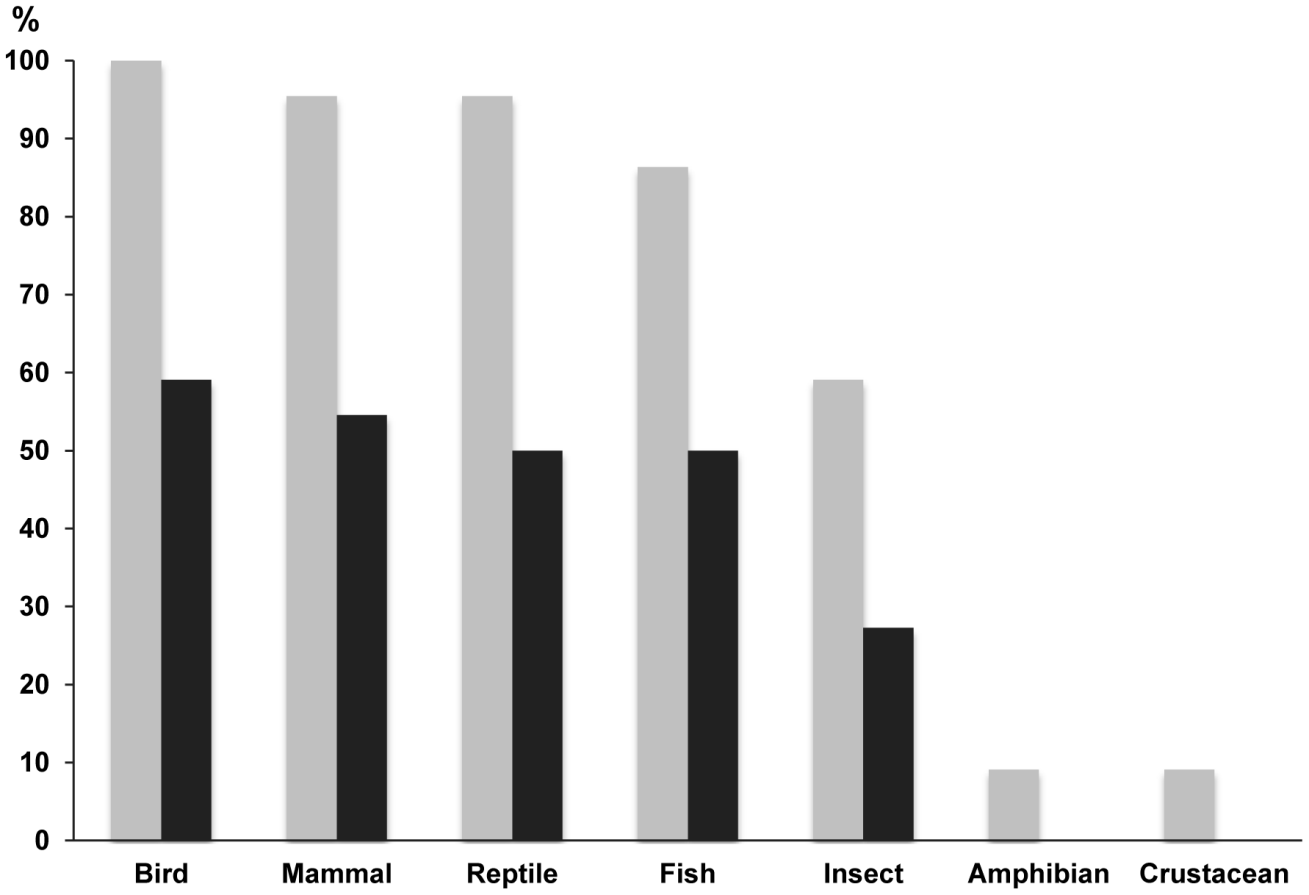
Relative frequency of wild animal taxa (%) between the present and future drawings. Frequency of presence of animal wildlife in drawings by children from 22 villages in Indonesian Borneo. Frequency of presence for each animal taxon is shown for present-day drawings (grey bars), and the future drawings (black bars).

Analysis of biodiversity features indicated that stark changes were expected in wild animal taxa, with declines in richness for all species groups (Figure 3) and in animal abundances between the present and future environments (p≤0.001, U = 7.5). (The presence of domestic animals did not show significant changes between present and future drawings.)

Similarly, children expected declines in botanical diversity (p≤0.05, U = 148.0), and in specific vegetation elements such as the number of resinous trees and number of different types of fruit trees. Grasslands (considered separately from other vegetation diversity) were often drawn in former forest areas in the future. Children often paid specific attention to the condition of wild animals: in the present they depicted ‘many animal species’ (77.3% of present drawings) and in some cases they indicated the animals were already threatened or very threatened (22.7%). In the future, the majority of the species disappear, with either few species persisting (22.7%) or no wild animals at all (40.9%). The drawings indicate that children expect an increase in the range of threats to animals (p<0.001, Cc = 0.676), with only two threats mentioned in the present (hunting and buildings, each in 5% of the drawings), but nine threats perceived for the future. For the future, three major threats were identified as arising from hunting, agriculture and forest fire, while several other threats also contributed to the disappearance of animals. The threats with highest frequencies in ‘future’ drawings consisted of urban development and logging for buildings (25%), agricultural development (20%), hunting (15%), agriculture and logging activities (15%), and a mix of these different threats (10%). Illegal logging was not drawn in the present, but was expected to occur in the future (35% of drawings, p≤0.05, Cc = 0.386).

The drawings indicate that environmental disasters are expected to become more frequent, with flood and landslide occurrence being significantly higher in the future (respectively: p = 0.019, Cc = 0.333; p = 0.008, Cc = 0.369; for the overall non-flood/landslide disasters: p = 0.019, Cc = 0.333). All elements involving development are also expected to increase, including the number of factories (timber and others, p = 0.019, U = 187.0), number of large buildings (p = 0.019, U = 187.0), presence and number of main roads (p = 0.004, respectively Cc = 0.398 and U = 152.5), and presence of industries (logging, mining, others; p = 0.031, Cc = 0.309). Finally, there was a clear expectation that active forest clearance will increase dramatically (p<0.001, U = 66.0). In the ‘present’ drawings, 40.9% showed ‘no clearing’, 31.8% showed ‘a few’ and 27.3% showed ‘many’ people clearing the forest. In contrast, all of the future drawings showed ‘many’ people clearing the forest.

### Physical landscape of villages

**Figure 4:**
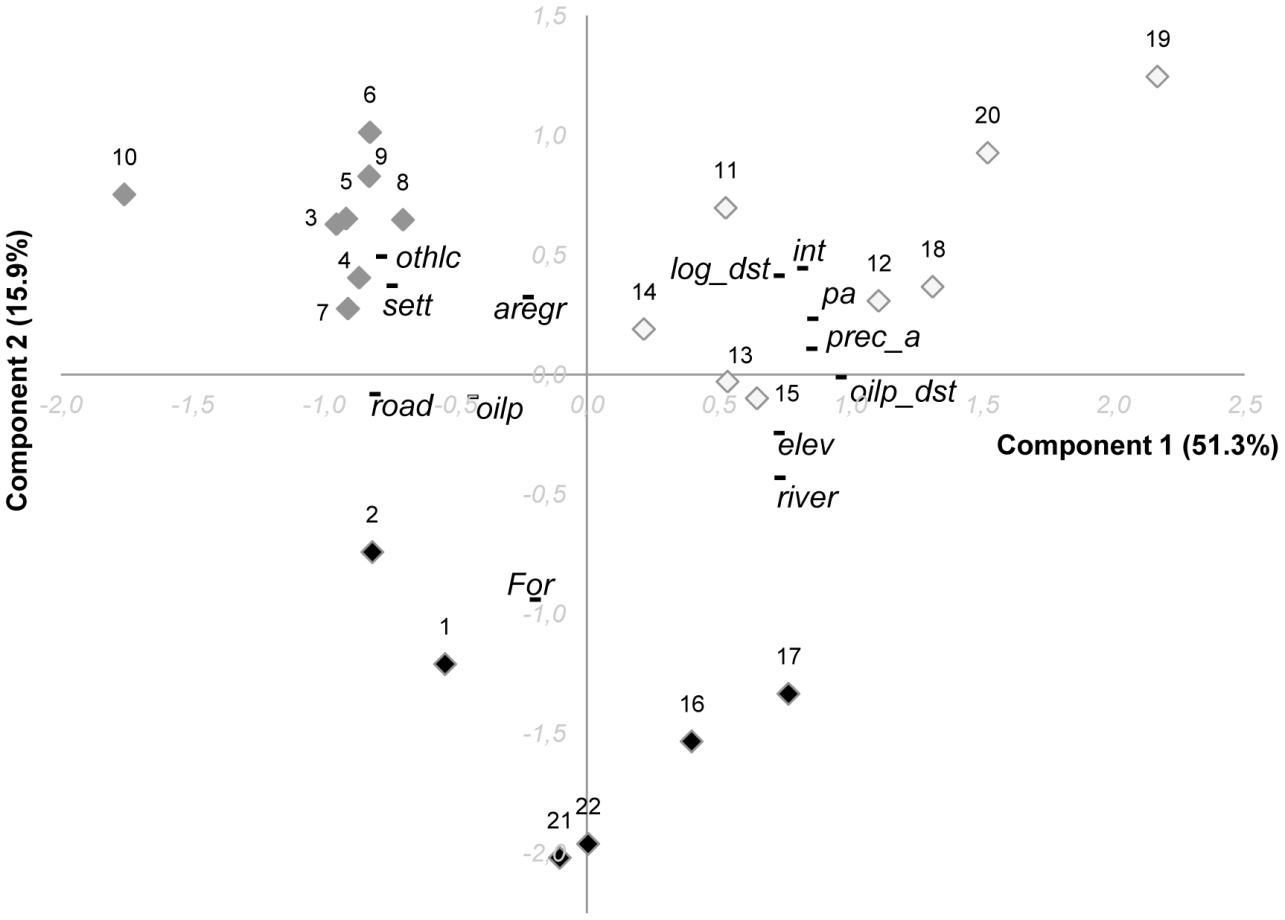
Principal component of the current land cover and land use data related to the villages. The figure shows the current physical landscapes of the villages. The first two components explain 67.2% of the variability of the data (respectively, component 1: 51.3% and component 2: 15.9%). Villages are numbered points (diamonds) and variables are shown as a short line (black). Villages with similar physical landscapes form a group charactherized in the figure by a specific color (white: villages 11, 13, 14, 15, 12, 18, 19 and 20; grey: villages 3, 4, 5, 6, 7, 8, 9 and 10; black: villages 1, 2, 16, 17, 21, and 22).

Figure 4 shows the relationships among land use and land cover variables for the areas surrounding the villages, as summarized by the principal component analysis (PCA). Principal component 1 captures 51.3% of the variation in the LULC data and the second principal component 15.9%. The PCA separates the villages into 3 groups: 1) a group consisting of eight villages from the upper Kapuas, particularly Kapuas Hulu District; 2) a group of eight villages from the lower Kapuas, particularly Pontianak District; and 3) a group consisting of two villages in East Kalimantan (further along component 2), two in upper Kapuas, and two in the lower Kapuas close to Pontianak district.

The first group is related to intact forest, protected area cover, annual precipitation, distances to logged forest and to oil palm, as well as river density and elevation. Four villages of this group are close to large protected areas (higher along component 1), with the latter three of these villages being close to a large national park and remote (especially 19 and 20). The second group is associated with other (non-forest) land covers, agroforest/regrowth and higher settlement density. Village 10 is rather set apart from this group along the component 1 due to its high values for oil palm plantations (oil palm land cover) and road density, and its lower values for distances to logged forests and to oil palm. The final group is composed of villages characterized by total forest cover (consisting mainly of logged rather than intact forest) and road density. The two villages from the lower Kapuas are also correlated with high oil palm coverage in their surroundings, while the villages from the upper Kapuas are slightly set apart in the PCA due to their relatively high values for river density and elevation.

### Multivariate analysis of art variables

**Figure 5:**
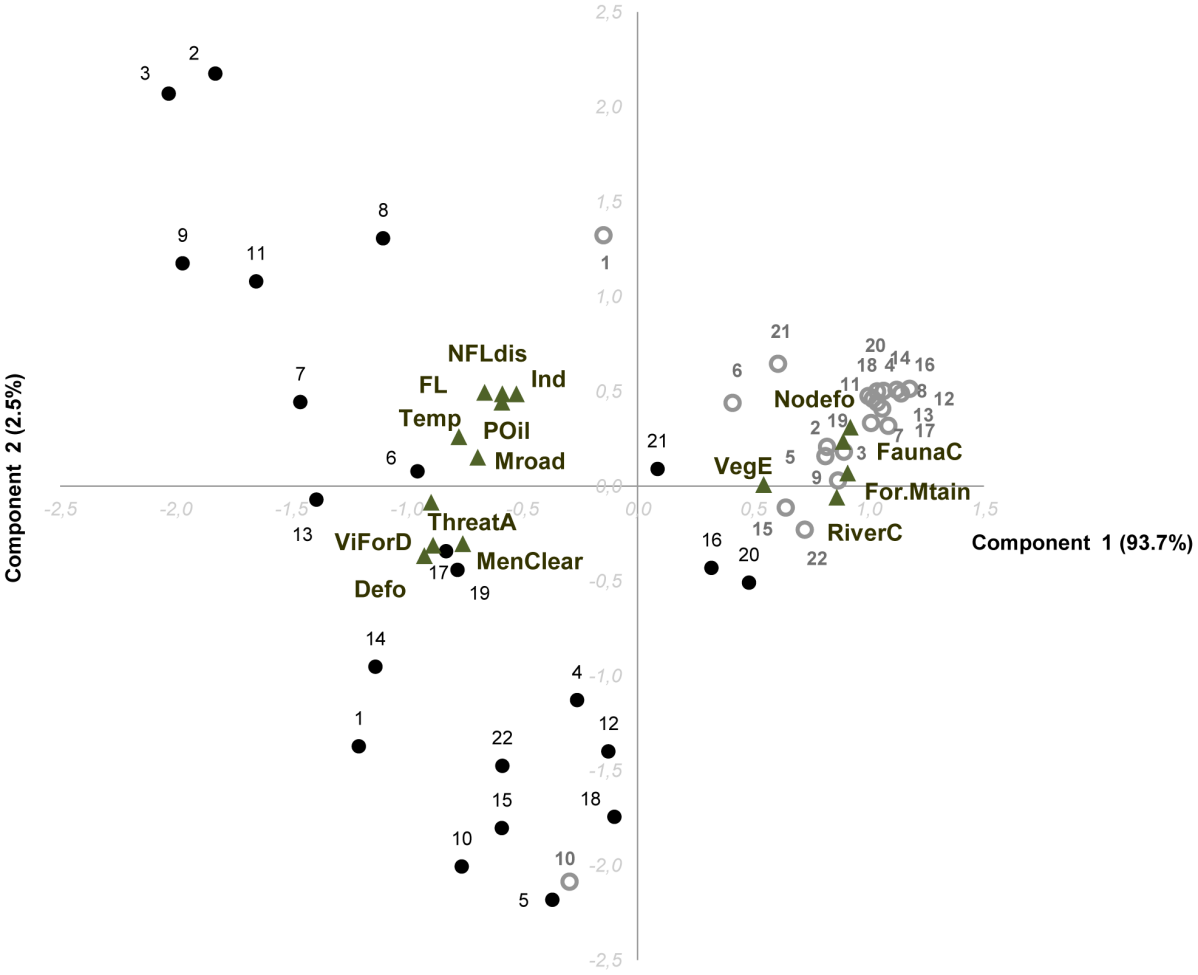
Categorical principal component of 15 art variables from ‘present’ and ‘future’ drawings. The figure shows the relationships between the art variables (green triangles; see Table 2 for their names and codes) and the villages from different landscapes (see Fig. 4) describing the children's perceptions of environmental change between the present (grey circles) and the future (black dots). The two first components explain 96.2% of the variability of the data (respectively, component 1: 93.7% and component 2: 2.5%).

A clear direction of change through time is visible in the positions of ‘present’ and ‘future’ variables in the drawings on the first two principal components in the CatPCA of the art variables (Figure 5), indicating consistent expectations about the direction of change in environmental conditions. The present drawings show better ‘conditions of mountain forests, river, fauna’, higher ‘vegetation diversity’, and less ‘deforestation and degradation’. In the future, children expect all these conditions to deteriorate, as shown by shifts along the first axis in a direction of more ‘floods and non-flood disasters’, higher ‘temperature’, more ‘disturbed areas’ with more ‘people clearing the forest’, and increases in ‘roads’, ‘industries’, ‘oil palm cover’, ‘threats to wildlife’ and ‘distance of the village to forest’. Two villages have ‘present’ conditions that are more degraded than other villages (visible as two grey dots outside the compact group of other villages, where grey represents conditions in the ‘present’ drawings). This separation from other villages is mainly due to the ‘presence of industries’ (varying along axis 2). Nonetheless, the children’s drawings from these villages show similar directions of future changes compared to other villages. There are no villages in which the children expected improved environmental conditions in the future (i.e., no black dots to the right of corresponding grey ones). Only three villages had ‘future’ conditions that were close to the main group of ‘present’ conditions which showed relatively good current conditions (high on axis 2) and relatively small declines into the future. All three were in the upper Kapuas.

It appears that the existing variation in landscape context across villages is expected to increase over time. Some patterns correspond to geographic regions, with villages (in the top quadrant) from the lower Kapuas (except one, no. 11, in the upper Kapuas), all being expected to suffer from higher ‘temperatures’, more environmental ‘disasters’, and to see more ‘industrial’, ‘main road’, and ‘oil palm’ developments. However, there is a very broad geographic spread among the villages (one from East Kalimantan, and the others from the two areas in West Kalimantan) that show future expectations of increased areas of ‘disturbed forest’, ’people clearing the forest’, and increased ‘village-forest distance’ and ‘threats to animals’.

The three villages with the largest expected changes between the present and future (as measured in the PCA by the Euclidean distance between the two periods for each village), are villages from the lower Kapuas area characterized by relatively high road and settlement densities, agro-forest/regrowth cover, other non-forested and oil palm land covers (i.e. high oil palm cover for village 1 and 2; and more rubber, coconut, cacao plantations, and oil palm plantations planning during 2012 for village 3). The two villages with the smallest expected changes between present and future consist of one village in a presently degraded area, where children expected slight further degradation into the future, and one village where children perceived good environmental conditions in the present only degrading slightly into the future. The first is a village in the lower Kapuas (village 10), which shows small changes from the degraded conditions of the present, to slightly further degraded conditions in the future. Current levels of degradation are also indicated in the drawing, and by the landscape variables for which it has the highest values of settlement and road densities, highest oil palm and other non-forested land covers, and lower distances to logged forests and to oil palm. The second village shows small changes from the generally good conditions of the present, and this is a village in East Kalimantan with the largest forest area (selectively logged natural forest), and lower settlement density (village 21).

### Correlation between land cover data and art variables

Several land cover variables are correlated with art variables in the present (see Table S4). Children living in landscapes with higher oil palm or other non-forest land covers drew pictures from the ‘present’ with larger ‘distance of the village to forest’ (respectively, r = 0.43 and 0.44, and p<0.05 in all cases mentioned). The land cover of oil palm plantations is also correlated with drawing of ‘main roads’ and ‘flood disasters’ (both resp., r = 0.52). Both oil palm land cover and road density are positively correlated with ‘threat to animals’ (resp., r = 1.0 and 0.52) and negatively correlated with ‘faunal condition’ (r = −0.73 for oil palm; −0.47 for road density). Total forest cover (characterized by more logged than intact forest) is positively correlated with ‘presence of industries’ (r = 0.44). Logged forest distance is negatively correlated with the number of ‘people clearing the forest’ (r = −0.47) and with the ‘distance of the village to forest’ (r = −0.58). The three physical layers of current river density, elevation and annual precipitation are negatively correlated with the art variable of ‘oil palm cover’ (resp., r = −0.59; −0.54 and −0.60).

Correlations between land cover variables and art variables in the future (see Table 3) show current intact forest cover and distance to oil palm negatively correlated with future ‘presence of main roads’ in drawings. On the contrary, this future art variable is positively correlated with current cover of oil palm plantations and road density. Children living in villages with lower forest cover drew greater ‘non-flood disasters’ while children living with larger cover of agroforest/regrowth surrounding their village drew more ‘flood disasters’. Other land cover, settlement density and agroforest/regrowth cover are negatively correlated with ‘undisturbed forest’ and ‘condition of mountain forests’. These first two landscape variables were also negatively correlated with the future ‘river condition’ and positively with ‘oil palm cover’ in drawings. Agroforest/regrowth and other land covers are also positively correlated with ‘disturbed forest areas’. Agroforest/regrowth cover is also associated with lower ‘faunal condition’. Distance to oil palm is negatively correlated with the art variable ‘oil palm cover’ in drawings of the future (though oil palm plantation cover is not). Other current landscape variables are correlated with ‘oil palm cover’ in future drawings, including negative correlations with protected area cover, river density and annual precipitation.

**Table 3:** Correlations between current land cover surrounding the village, and art variables representing children's drawings of their future environment.

		Intact forest	Logged forest distance	Forest cover	Agroforest / regrowth	Oil palm plantations	Oil palm distance	Other land cover	Protected areas	River density	Elevation	Settlements	Road density	Annual precipitation
Temperature condition	*Corr.*	−.23	−.02	.23	.01	.07	−.37	.12	−.33	−.10	−.18	.13	.29	−.17
Forest Mountain condition	*Corr.*	.18	.08	.05	−.68**	−.28	.60**	−.68**	.58**	−.09	.65**	−.65**	−.36	.12
River Condition	*Corr.*	.11	.07	.11	−.45	−.32	.44	−.59**	.37	.03	.38	−.54*	−.21	.22
Village-Forest Distance	*Corr.*	−.05	.01	−.05	.26	.27	−.34	.32	−.31	.09	−.35	.26	.24	−.01
Undisturbed forest	*Corr.*	.09	.19	.11	−.44*	−.24	.52*	−.65**	.55**	.10	.63**	−.53*	−.35	.17
Disturbed forest	*Corr.*	.06	.02	−.15	.46*	.35	−.40	.47*	−.35	.23	−.41	.35	.22	.09
Oil palm area cover	*Corr.*	−.22	−.19	−.01	.22	.09	−.47*	.57**	−.43*	−.53*	−.40	.47*	.38	−.51*
Industries	*Corr.*	−.20	−.11	.22	.08	−.04	−.25	.04	−.28	−.10	−.05	.04	.22	−.13
Floods	*Corr.*	.06	.28	−.22	.46*	.00	−.30	.31	−.39	−.02	−.31	.24	.08	.02
Main road	*Corr.*	−.46*	−.24	.17	.26	.48*	−.55**	.39	−.40	−.02	−.31	.34	.47*	−.16
Faunal Condition	*Corr.*	.14	.09	−.11	−.65**	−.26	.37	−.39	.28	−.27	.08	−.29	−.26	−.06
Vegetation diversity	*Corr.*	.03	−.13	.15	−.28	−.28	.32	−.29	.25	−.11	.19	−.22	−.28	.09
Non−Flood disasters	*Corr.*	.18	.25	−.45*	.17	.00	−.22	.32	−.17	−.27	−.28	.30	.07	−.25
Threats to Animals	*Corr.*	−.08	−.21	.06	.24	−.27	−.05	.34	−.10	−.37	−.08	.20	.02	−.34

For descriptions of land cover variables and of art variables, see respectively Table S1 and Table 2. The art variable ‘People clearing the forest’ has no variance for the future, with the highest number of men clearing the forest for each village, its correlation with the land cover is thus not calculated. Correlation coefficients shown in bold font are statistically significant (with *p<0.05 and **p<0.005).

### Multivariate analysis of art and land cover variables: present-day context

**Figure 6:**
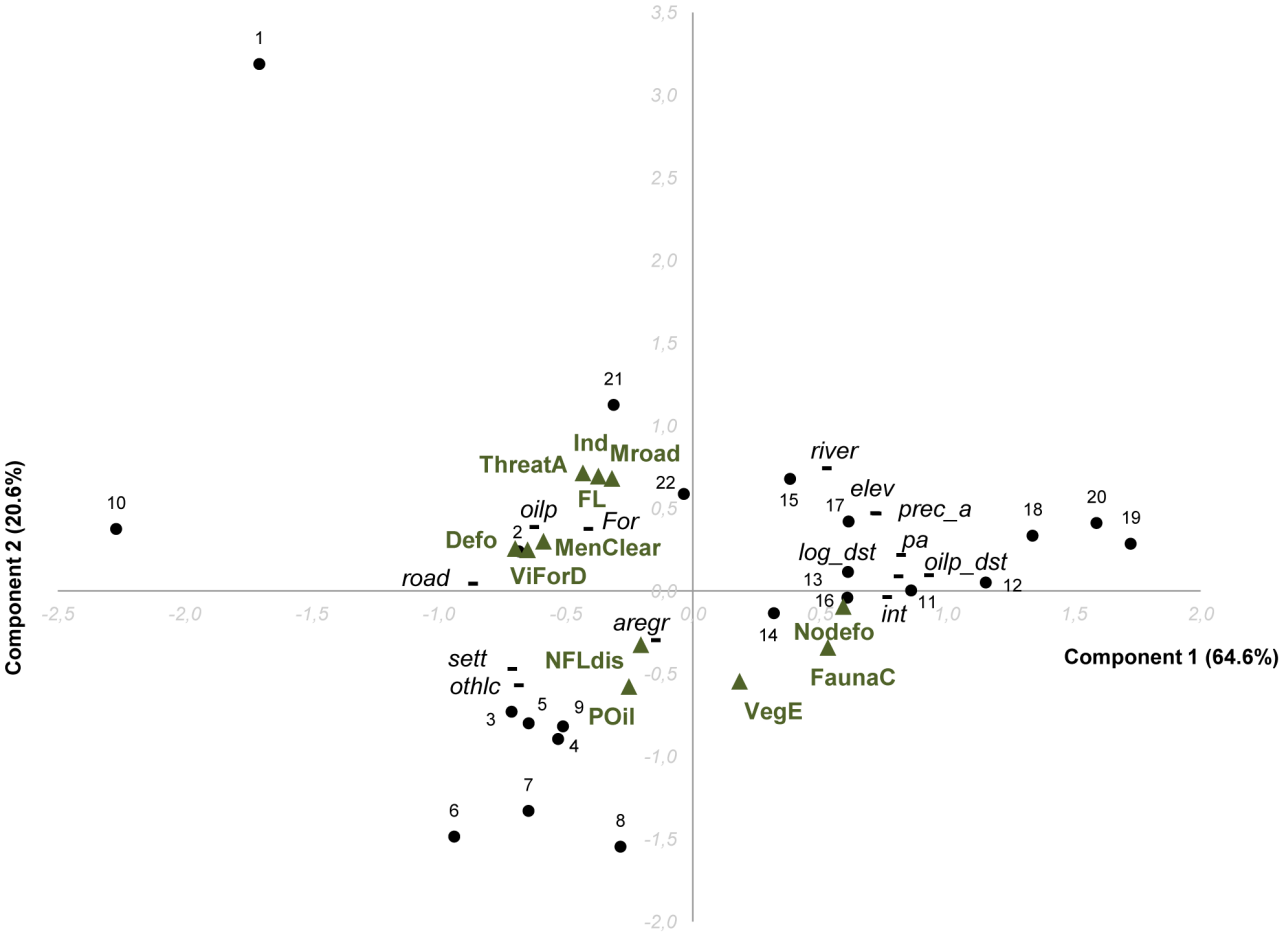
Categorical principal component of the current land cover, and art variables of the ‘present’ environment. The figure shows the relationships among the current land cover (black short line) of the villages (black dots) and the art variables from drawings (green triangles; see Table 2 for their names and codes) describing the children's perceptions of current environmental conditions. The two first components explain 85.2% of the variability of the data (respectively, component 1: 64.6% and component 2: 20.6%) and separate three village groups (Group 1: high values along axis 1, consisting of villages 11, 13, 14, 15, 16, 17, 12, 18, 19 and 20; Group 2: low values on axis2, consisting of villages 3, 4, 5, 6, 7, 8 and 9; Group 3: relatively low values on axis 1, consisting of villages 1, 2, 21, 22 and 10). The art variables ‘temperature, forest mountain, and river conditions’ have no variance in the present-day context across villages (perceived in ‘good condition’), and were thus not included in the PCA.

Figure 6 shows the multivariate relationships between the current landscape context of the villages where the children are living, and their perceptions of current environmental conditions (bivariate correlations between land cover and art variables were presented previously in Table S4). The two first components explain 85.2% of the variability of the data (respectively, component 1: 64.6% and component 2: 20.6%). The villages separate into 3 groups (Figure 6). Group 1 consists of ten villages with high values on axis 1; Group 2 of seven villages with low values on axis 2; and Group 3 of five villages with relatively low values on axis 1.

Group 1 consists of ten villages from the upper Kapuas, including the three remote villages close to a national park, associated with high values for several physical landscape layers: protected area cover, intact forest cover, larger distances to oil palm plantations and to logged forest, higher river density, elevation and annual precipitation. Drawings from these villages showed more ‘undisturbed forest areas’, higher ‘total vegetation diversity’, and better ‘faunal condition’.

Group 2 consists of seven villages in the lower Kapuas region (Pontianak district), that have higher settlement densities and other non-forest land cover, and in which children drew more ‘oil palm areas’, and ‘non-flood disasters’.

Group 3 consists of two remote villages in East Kalimantan, along with three villages in the lower Kapuas. These villages show moderate forest cover (dominated by logged forest), and the three villages in the lower Kapuas also have higher oil palm land cover and road density (showing the highest values for these variables). The village from Pontianak district is further apart along axis 1 also due to its high value for other non-forest land cover and highest value for settlement density. Drawings from children in this group of villages showed more frequent occurrences of ‘industries’, ‘main roads’ and of ‘floods’, and higher ‘disturbed forest areas’, ‘village-forest distance’ and greater ‘threats to animals’ and ‘people clearing the forest’.

### Multivariate analysis of art and land cover variables: current landscape, future drawings

**Figure 7:**
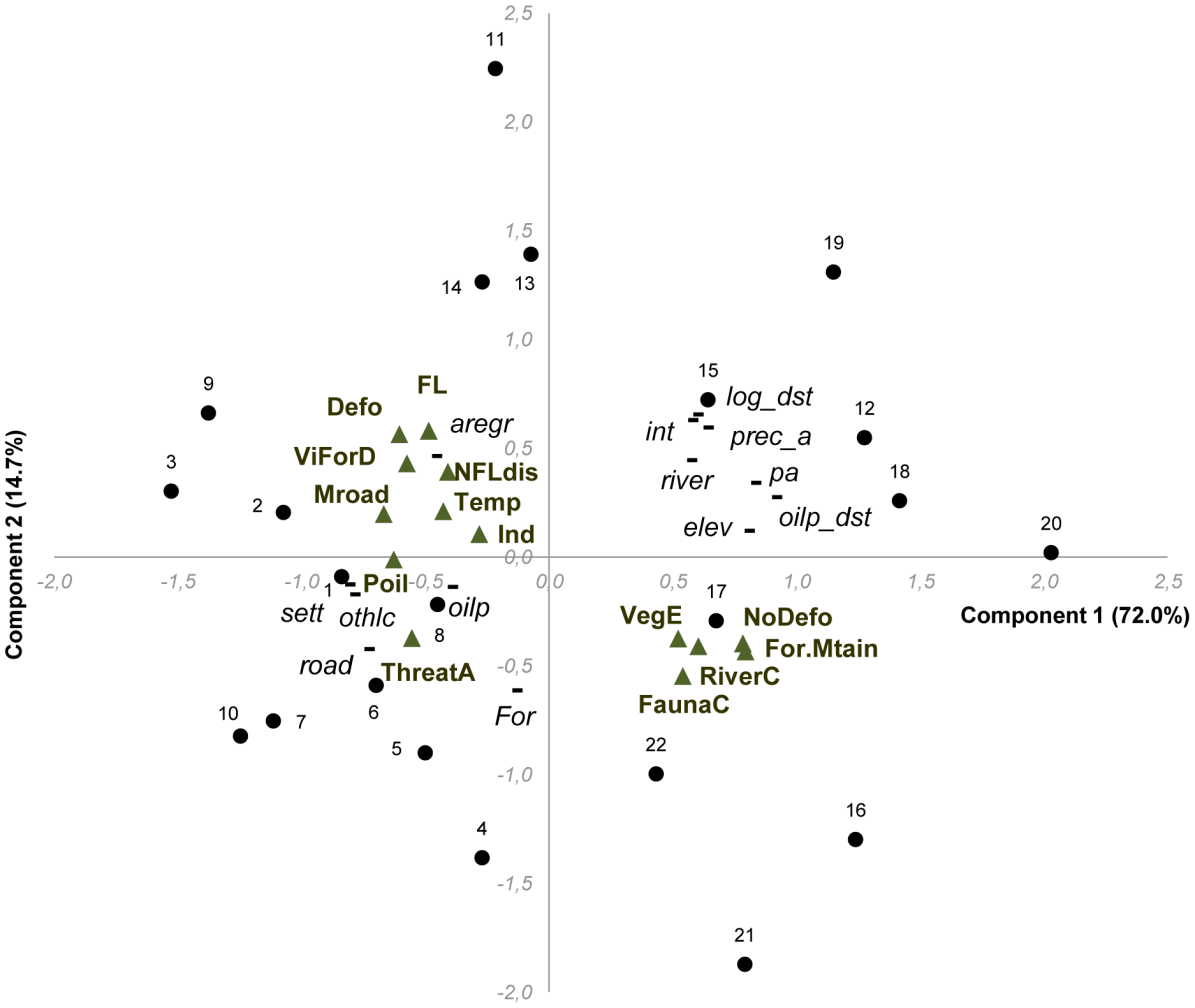
Categorical principal component of current land cover, and art variables of the ‘future’ environment. The figure shows the relationships between the current land cover (black short line) of the villages (black dots) and the art variables from drawings (green triangles; see Table 2 for their names and codes) describing the children's perceptions of environmental conditions for the future, in 15 years’ time. The two first components explain 86.8% of the variability of the data (respectively, component 1: 72.0% and component 2: 14.7%). The villages fall into three broad groups (Group 1: high values on axis 1, consisting of villages 15, 16, 17, 12, 18, 19, 20 and 21, 22; Group 2: low values on axis 1, consisting of villages 2, 3, 9, 11, 13 and 14; Group 3: low values on axis 2, consisting of villages 1, 4, 5, 6, 7, 8, and 10. The art variable ‘People clearing the forest’ was high for all villages, and thus has no variance across villages and was not included in the PCA.

Figure 7 shows the multivariate relationships between the current landscape context of the villages where the children are living, and their perceptions of environmental conditions in the future (bivariate correlations were presented in the Table 3). The two first components explain 86.8% of the variability of the data (respectively, component 1: 72.0% and component 2: 14.7%). Generally the villages separate into 3 groups: 1) a group consisting of the two remote villages in East Kalimantan, along with seven villages in the upper Kapuas (including the three close to a national park (18, 19 and 20)); 2) a group of three villages from the lower Kapuas and three from the upper Kapuas; and 3) a group of seven villages in the lower Kapuas, within or near Pontianak district.

Village group 1 is correlated with the art variables for future conditions of ‘river’, ‘animals’, ‘mountain forests’, the ‘undisturbed forest areas’ and ‘vegetation diversity’. This group is correlated with the physical land cover of intact forest, protected areas, river density, elevation, and annual precipitation, and, to some extent, distances to logged forest and to oil palm plantations.

Village group 2 is largely associated with future drawings of worse ‘temperature condition’, greater occurrences of ‘floods’ and other ‘natural disasters’, ‘presence of industries’ and ‘main roads’, higher ‘village-forest distance’ and areas of ‘disturbed forest’. Three villages in the upper Kapuas are higher along axis 2 due to relatively highest values of agroforest/regrowth cover and their association with ‘disturbed forest’. This group is correlated with higher current agroforest/regrowth cover, explained in both components.

Village group 3 is correlated with the art variables of future ‘oil palm cover’, and ‘threats to animals’, and the current physical land covers of oil palm plantations, other non-forest land cover, settlements and road densities (along axis 1) and total forest cover (along axis 2).

## Discussion

This study confirms that drawings provide powerful tools to gain insights into children’s thoughts about forests, wildlife and environmental change [25], [26]. Verbal explanations from children about their drawings were an important complement to the drawings themselves, as suggested by Malchiodi [27]. Similar to findings by Alerby [25], in the present study young children were actively engaged in thinking about their environment, and were able to convey their thoughts through the symbolic language of their drawings.

Our results indicate that children have sophisticated perceptions of their direct environments, including general conditions of forests and rivers, fauna and flora, and human activities; and have an understanding of how these variables influence each other. Indonesia's rich biodiversity is being rapidly degraded by landscape change, pollution and overharvesting. The children in this study show a strong awareness of these issues, and generally share similar perceptions of their environment and the causes and consequences of environmental degradation, along with an understanding of human needs and environmental protection. They comprehend the impacts of human activities on wildlife and other natural resources, via land use change and landscape fragmentation, and also through increasing natural disasters and temperature.

Our results indicate that children have landscape context-dependent perceptions of current and future environmental conditions, which may be influenced by awareness about past environmental conditions. Children's perceptions often depend strongly on their previous and current experiences in specific contextual conditions [18]. Our study proves that this is the case for rural children in Borneo who have consistent perceptions of their current environment and expectations of deteriorating future environmental conditions that both reflect the landscape that currently surrounds them. As long as forest cover remains high, the views of the current environment and its expectation are that the atmosphere, rivers and wildlife continue to be in relatively good condition, although some other aspects of the environment are still expected to decline in the future. In contrast, in areas where limited forest remains or forests are heavily degraded due to human activities, the current environment is perceived with similarly high awareness, but the expectation is of much faster, ‘run-away’ transition to non-forest landscapes and a collapse of natural environmental functions. Moreover, it appears that the more the children have been exposed to land use and environmental change, the more they expect such trends to continue and to worsen in the future (e.g. increase in infrastructure, climate change and natural disasters).

The strong associations that our study found between present and future environmental conditions indicate that there is no obvious shifting baseline among Kalimantan's children regarding perceptions of their environment. Children expressed strong perceptions of how present and future land uses are entwined. Most children predicted future environmental conditions to be worse than the present, suggesting an insight into past environmental trends. If either generational [2] or personal amnesia [28] existed, a more likely outcome would have been that children would make similar predictions about their future environmental conditions, irrespective of how current conditions had been caused by past processes. In our present study, this was not the case. Clearly, a proper test of shifting baselines requires consideration of older generations as well as children. The shifting baseline syndrome would assume that adults have a more accurate understanding of past environmental conditions and trends than children. Because these trends are negative (from a forest conservation point of view), adults would be expected to have more negative environmental expectations than children who would then perceive current and probable future land use patterns as a normal condition [7]. As part of our broader research program, we did assess the perceptions of adults about the past, present and future condition of their village environments, but using a different approach, namely the pebble distribution method [29]. Preliminary analysis of the adult's perceptions indicate that, similar to children, adults perceive deteriorating environmental conditions in their present village environments, and see the causes of present-day conditions as likely to continue and worsen into the future (ASP, unpubl. data). Children and adults expressed qualitatively similar predictions of future environmental conditions (especially an increase in temperature, pollution and flood frequencies (for adult perceptions of floods, see [17]). However, we cannot assess these similarities quantitatively, due to the different methods of collecting information.

The apparent absence of a shifting environmental baseline among Kalimantan's children may be explained by diverse informal and formal learning processes that shape children's perception on current and future environmental conditions. Testing of these factors is beyond the scope of this paper, however we note that children in the most remote and forested areas, where conservation education at school was noted to be rare during the present study, often attend village meetings that involve discussion of natural resources and decisions on land use management. We noted that during such meetings, experiences from other nearby villages were often discussed to inform the villagers' own decision-making. For example, if oil palm plantations are expanding in neighbouring village areas, many stories are discussed about land prices, labour opportunities, local access to forest resources, or conflicts with companies or local governments. Children's participation in such informal meetings likely forms a strong basis for their environmental awareness. In addition, it is likely that the more communities depend on the use of forests, forest gardens, and other natural resources, and the more children within such communities engage in these processes, the more they understand about their environment and connection to nature, and the more aware they would be of the changes that might occur in their direct environment. For example, communities that depend for much of their animal protein on fish and wild meat such as pigs and deer are highly aware of the temporal and spatial trends in populations of their target species. Communities that instead have livelihoods based mainly on income from agriculture or from employment in timber, mining, or plantation industries would be expected to have lower awareness or reduced sensitivity to environmental trends. However, this was not apparent in the current study, since children in already-degraded areas did express awareness of current environmental problems, and greater expectations of negative changes in the future than did children in more intact forest landscapes.

Finally, children's perceptions on deteriorating environmental conditions across different landscapes may be shaped by the provision of environmental courses at school, or the availability of a range of media (including books, television and radio). National and international information on environmental degradation is generally negative and focuses on declining populations of endangered species (e.g., orangutans), deforestation, or increases in environmental disasters (e.g., flooding). Children living in more remote areas (usually with more intact forests) may have less access to such information compared to children in more degraded landscapes and so may hold views of their current and future environments that are less influenced by the generally negative trends portrayed in media. These different forms of learning and sources of information are not mutually exclusive and are likely to interact, depending on the natural and social context in which the children are living. Further studies would be needed to differentiate how diverse sources of information affect environmental thinking among children in different parts of tropical landscapes. This would involve assessing differences in the accessibility of environmental education and other information sources, and the time children spend in forests in different multifunctional landscapes. Understanding which factors and information sources most strongly affect environmental opinions would help design more effective methods for increasing environmental awareness.

A further message from our study for forest conservation and sustainable resource management is that a large shift in practices and perceptions is needed to alter the current trajectory of change, and avert severe losses of forest and wildlife. As most children foresee a future of further environmental degradation, it is essential to develop approaches to show these children that past trends do not necessarily determine future trends, and that positive changes are possible if development actively considers biodiversity and dependence of communities on ecosystem services. The strong environmental awareness among children in Kalimantan provides a good starting point for educational programs and their support for conservation of biodiversity and ecosystem services. Awareness of trade-offs involved in forest conservation and conversion to different land uses will enable children to value the benefits from the environment and to make more informed decisions later in their lives when they participate in decision making. In that regard, it is important for children to start learning about nature as early as possible, especially in view of the high sensitivity of children to stereotypical imaging such as they encounter in the media and literature [30]. Education, environmental experience, media and other types of communication could thus play major roles in promoting support among younger generations for more sustainable environmental management, and the children's drawings themselves could play a useful role in this. Indeed, the strong story-telling potential of the drawings could provide a powerful tool by orienting NGOs and educational practitioners in educational initiatives to regions where conservation awareness is the most needed. Also the drawings could be used to influence political thinking and decision-making on land use, particularly in democratic governments where citizens' views hold considerable influence in public policy. Children have the highest stake in ensuring that land use maximizes their long-term well-being, and their views and aspirations should thus be seriously considered by local and international political decision-makers elected to represent the citizens' voices and fulfill efficient natural resource management.

## Supporting Information

Table S1 **Summary of the 13 spatial predictor variables used in the analysis.** For processing steps refer to [8].
(DOC)

Table S2 **Correlations between art variables in the set of present-day drawings.**
(DOC)

Table S3 **Correlations between art variables in the set of future drawings.**
(DOC)

Table S4 **Correlations between the present land cover surrounding the village, and art variables from drawings of the present environmental conditions.**
(DOC)
