# Supplementary material for: Through the Eyes of Children: Perceptions of Environmental Change in Tropical Forests
Source: PLoS One. 2014 Aug 5;9(8):e103005. doi: 10.1371/journal.pone.0103005 (PMC4122389; doi:10.1371/journal.pone.0103005)
Supplement: Table S1 — Summary of the 13 spatial predictor variables used in the analysis. For processing steps refer to [8]. (DOC) [file pone.0103005.s001.doc]

**Through the eyes of children: Perceptions of environmental change in tropical forests**

**Table S1**. Summary of the 13 spatial predictor variables used in the analysis. For processing steps refer to [8].

| **General class** | **Predictor variables (abbreviations)** |
| --- | --- |
| **Land Use Land Cover** | **Intact forest (int) - S *^1^**  Medium to tall old-growth natural forests that have never been logged by the timber industry. Open to closed canopy: closure is probably higher than 30%. Includes Lowland and montane Dipterocarp forests, riverine forests, heath forests on plateaux, tall closed-canopy peat forests and open-canopy pole peat forests. Note, our intact forest class may include areas where the forest has been degraded slightly by small-scale logging, which we could not detect using Landsat imagery. |
|  | **Logged forest (log_dst) - m *^2^**  Medium to tall old-growth natural forests that have been logged by the timber industry using heavy machinery and networks of logging trails. Open to closed canopy: Includes Lowland and montane Dipterocarp forests and tall closed-canopy peat forests. |
|  | **Forest (For) - S**  Represents both intact natural forest (see above) and logged forest (see above), as a combined layer. |
|  | **Agro-forest or forest regrowth (aregr) - S**  Medium to tall agro-forests and forest regrowth. Open to closed canopy: closure is probably equal or higher than 30%. Includes traditional rubber agroforests, fruit gardens, and land under fallow, where forests is regenerating. |
|  | **Oil palm plantations in 2010 - S (oilp) and m (oilp_dst)**  Planted or recently cleared industrial scale oil palm plantations as of year 2010. |
|  | **Other land cover (non-forest; othlc) - S**  Includes: 1) Low vegetation of grasses or shrubs occurring on drained soils, occasionally flooded; 2) dry rice cultivation; 3) Low herbaceous vegetation with including tall grasslands and ferns; 4) can include agricultural cropland areas; 5) dry to occasionally flooded terrain; 6) areas of herbaceous vegetation, 7) shrub lands and young forest regrowth in fallow lands. |
|  | **Protected areas (pa) - S**  All protected areas such as natural reserves, other protected forests and National Parks. |
| **Topography** | **Elevation above sea level** in meters **(elev)** |
|  | **River density** in km^2^ **(river)** |
| **Climate** | **Annual precipitation** in mm **(prec_a)** |
| **Infrastructure** | **Road density** within 5 km radius **(road)** |
|  | **Settlement density** within 10 km radius **(sett)** |

*^1^  S = summed cover in all cells within a radius of 10 km from the village or cell centre-point.

*^2^ m = distance in meters to the nearest example of the land cover, from the village or cell centre-point.
