# Supplementary material for: Through the Eyes of Children: Perceptions of Environmental Change in Tropical Forests
Source: PLoS One. 2014 Aug 5;9(8):e103005. doi: 10.1371/journal.pone.0103005 (PMC4122389; doi:10.1371/journal.pone.0103005)
Supplement: Table S2 — Correlations between art variables in the set of present-day drawings. (DOC) [file pone.0103005.s002.doc]

**Through the eyes of children: Perceptions of environmental change in tropical forests**

**Table S2**. Correlations between art variables in the set of present-day drawings. Correlation coefficients shown in bold font are statistically significant (with *p < 0.05 and **p < 0.005). The art variables 'Temperature (Temp), Forest Mountain (For.Mtain) and River (RiverC) conditions' have no variance in the present-day context (they were perceived in ‘good condition’ in all villages), and their correlations with the other art variables are thus not calculated.

|  |  | Village-Forest Distance | Undisturbed forest | Disturbed forest | Oil palm area cover | People Clearing forest | Industries | Floods | Main road | Faunal Condition | Vegetation diversity | Non-Flood disasters |
| --- | --- | --- | --- | --- | --- | --- | --- | --- | --- | --- | --- | --- |
| Undisturbed forest | *Corr.* | -.34 |  |  |  |  |  |  |  |  |  |  |
| Disturbed forest | *Corr.* | **.43^*^** | **-.82^**^** |  |  |  |  |  |  |  |  |  |
| Oil palm area cover | *Corr.* | -.10 | .02 | .00 |  |  |  |  |  |  |  |  |
| People Clearing the forest | *Corr.* | .35 | **-.48^*^** | **.73^**^** | .26 |  |  |  |  |  |  |  |
| Industries | *Corr.* | .23 | -.07 | .41 | -.13 | **.43^*^** |  |  |  |  |  |  |
| Floods | *Corr.* | .16 | -.27 | .28 | -.09 | .29 | **.69^**^** |  |  |  |  |  |
| Main road | *Corr.* | .16 | -.27 | .28 | -.09 | .29 | **.69^**^** | **1.00^**^** |  |  |  |  |
| Faunal Condition | *Corr.* | -.29 | .30 | -.33 | -.09 | -.23 | -.21 | -.40 | -.40 |  |  |  |
| Vegetation diversity | *Corr.* | **-.50^*^** | **.47^*^** | **-.64^**^** | .31 | **-.46^*^** | **-.44^*^** | -.35 | -.35 | .21 |  |  |
| Non-Flood disasters | *Corr.* | .32 | -.27 | .28 | **.61^**^** | .29 | -.07 | -.05 | -.05 | .12 | -.10 |  |
| Threats to Animals | *Corr.* | .30 | -.12 | .14 | -.14 | .04 | .42 | **.65^**^** | **.65^**^** | **-.79^**^** | -.14 | -.08 |
