# Supplementary material for: Through the Eyes of Children: Perceptions of Environmental Change in Tropical Forests
Source: PLoS One. 2014 Aug 5;9(8):e103005. doi: 10.1371/journal.pone.0103005 (PMC4122389; doi:10.1371/journal.pone.0103005)
Supplement: Table S3 — Correlations between art variables in the set of future drawings. (DOC) [file pone.0103005.s003.doc]

**Through the eyes of children: Perceptions of environmental change in tropical forests**

**Table S3.** Correlations between art variables in the set of future drawings. Correlation coefficients in bold font are statistically significant (with *p < 0.05 and **p < 0.005). The art variable ‘people clearing the forest’ (MenClear) has no variance (because all villages depicted the highest level of the variable) and so its correlation with each of the other art variables is thus not calculated.

|  |  | Temperature condition | Forest Mountain condition | River Condition | Village-Forest Distance | Undisturbed forest | Disturbed forest | Oil palm area cover | Industries | Floods | Main road | Faunal Condition | Vegetation diversity | Non-Flood disasters |
| --- | --- | --- | --- | --- | --- | --- | --- | --- | --- | --- | --- | --- | --- | --- |
| Forest Mountain condition | *Corr.* | -.45 |  |  |  |  |  |  |  |  |  |  |  |  |
| River Condition | *Corr.* | -.41 | **.75^**^** |  |  |  |  |  |  |  |  |  |  |  |
| Village-Forest Distance | *Corr.* | .32 | **-.57^*^** | -.41 |  |  |  |  |  |  |  |  |  |  |
| Undisturbed forest | *Corr.* | -.34 | **.89^**^** | **.63^**^** | **-.61^**^** |  |  |  |  |  |  |  |  |  |
| Disturbed forest | *Corr.* | .28 | **-.85^**^** | **-.54^*^** | **.65^**^** | **-.82^**^** |  |  |  |  |  |  |  |  |
| Oil palm area cover | *Corr.* | .29 | -.44 | **-.47^*^** | .16 | **-.43^*^** | .04 |  |  |  |  |  |  |  |
| Industries | *Corr.* | **.66^**^** | -.16 | -.15 | .15 | -.16 | -.02 | .27 |  |  |  |  |  |  |
| Floods | *Corr.* | **.45^*^** | **-.65^**^** | **-.47^*^** | **.46^*^** | **-.46^*^** | **.48^*^** | .33 | **.50^*^** |  |  |  |  |  |
| Main road | *Corr.* | .33 | **-.68^**^** | **-.46^*^** | **.56^**^** | **-.64^**^** | **.56^**^** | **.43^*^** | .14 | **.42^*^** |  |  |  |  |
| Faunal Condition | *Corr.* | -.27 | **.72^**^** | **.63^**^** | -.29 | **.58^**^** | **-.59^**^** | -.33 | -.27 | **-.48^*^** | **-.50^*^** |  |  |  |
| Vegetation diversity | *Corr.* | -.30 | .34 | .33 | **-.69^**^** | .40 | **-.45^*^** | -.21 | -.19 | **-.45^*^** | **-.49^*^** | .32 |  |  |
| Non-Flood disasters | *Corr.* | **.45^*^** | -.35 | **-.47^*^** | .27 | -.31 | .15 | **.56^*^** | .30 | **.58^**^** | .23 | -.10 | **-.45^*^** |  |
| Threats to Animals | *Corr.* | -.03 | -.28 | -.34 | .06 | -.24 | -.06 | **.74^**^** | .17 | .20 | .26 | -.37 | .09 | .24 |
