# Supplementary material for: Through the Eyes of Children: Perceptions of Environmental Change in Tropical Forests
Source: PLoS One. 2014 Aug 5;9(8):e103005. doi: 10.1371/journal.pone.0103005 (PMC4122389; doi:10.1371/journal.pone.0103005)
Supplement: Table S4 — Correlations between the present land cover surrounding the village, and art variables from drawings of the present environmental conditions. (DOC) [file pone.0103005.s004.doc]

**Through the eyes of children: Perceptions of environmental change in tropical forests**

**Table S4.** Correlations between the present land cover surrounding the village, and art variables from drawings of the present environmental conditions. For descriptions of land cover variables and of art variables, see respectively Table S1 and Table 2. Correlation coefficients shown in bold font are statistically significant (with *p < 0.05 and **p < 0.005). The art variables 'Temperature (Temp), Forest Mountain (For.Mtain) and River (RiverC) conditions' have no variance for the present, perceived in ‘good condition’ across all villages, their correlations with the other art variables are thus not calculated.

|  |  | Intact forest | Logged forest distance | Forest cover | Agroforest / regrowth | Oil palm plantations | Oil palm distance | Other land cover | Protected areas | River density | Elevation | Settlements | Road density | Annual precipitation |
| --- | --- | --- | --- | --- | --- | --- | --- | --- | --- | --- | --- | --- | --- | --- |
| Village-Forest Distance | *Corr.* | -.32 | **-.58**** | .28 | .15 | **.43*** | -.41 | **.44*** | -.41 | -.05 | -.13 | .29 | .42 | -.31 |
| Undisturbed forest | *Corr.* | .23 | .30 | -.06 | -.04 | -.33 | .28 | -.24 | .14 | .03 | .24 | -.35 | -.38 | .20 |
| Disturbed forest | *Corr.* | -.30 | -.39 | .24 | -.18 | .35 | -.23 | .05 | -.16 | .05 | -.06 | .08 | .40 | -.20 |
| Oil palm area cover | *Corr.* | .00 | -.31 | -.17 | -.03 | -.16 | -.15 | .41 | -.32 | **-.59**** | **-.54**** | .35 | .09 | **-.60**** |
| People Clearing forest | *Corr.* | -.27 | **-.47*** | .21 | -.30 | .23 | -.33 | .24 | -.27 | -.21 | -.21 | .07 | .38 | **-.45*** |
| Industries | *Corr.* | -.34 | -.36 | **.44*** | -.32 | .31 | -.22 | -.19 | -.25 | .22 | .12 | -.21 | .35 | -.12 |
| Floods | *Corr.* | -.23 | -.25 | .26 | -.09 | **.52*** | -.29 | .02 | -.17 | .27 | -.09 | .05 | .29 | .02 |
| Main road | *Corr.* | -.23 | -.25 | .26 | -.09 | **.52*** | -.29 | .02 | -.17 | .27 | -.09 | .05 | .29 | .02 |
| Faunal Condition | *Corr.* | .40 | .29 | -.33 | -.15 | **-.73**** | **.44*** | -.26 | .22 | -.07 | .22 | -.33 | **-.47*** | .10 |
| Vegetation diversity | *Corr.* | .03 | .18 | -.03 | -.10 | -.27 | .13 | -.00 | .06 | -.36 | -.05 | .05 | -.23 | -.14 |
| Non-Flood disasters | *Corr.* | .16 | -.25 | -.22 | .05 | -.09 | -.05 | .30 | -.17 | -.30 | -.29 | .22 | .05 | -.36 |
| Threats to Animals | *Corr.* | -.36 | -.18 | .31 | .08 | **1.00**** | **-.52*** | .06 | -.26 | .23 | -.02 | .15 | **.52*** | -.00 |
